# Semen-Derived Exosomes Mediate Immune Escape and Transmission of Reticuloendotheliosis Virus

**DOI:** 10.3389/fimmu.2021.735280

**Published:** 2021-10-01

**Authors:** Qi Su, Yawen Zhang, Zhizhong Cui, Shuang Chang, Peng Zhao

**Affiliations:** ^1^ College of Veterinary Medicine, Shandong Agricultural University, Tai’an City, China; ^2^ Shandong Provincial Key Laboratory of Animal Biotechnology and Disease Control and Prevention, Tai’an City, China; ^3^ Shandong Provincial Engineering Technology Research Center of Animal Disease Control and Prevention, Tai’an City, China

**Keywords:** cock semen, exosome, reticuloendotheliosis virus, transmission, immune escape

## Abstract

Reticuloendotheliosis virus (REV) causes immune-suppression disease in poultry, leading to a significant economic burden worldwide. Recent evidence demonstrated that the REV can enter the semen and then induce artificial insemination, but how the virus gets into semen was little known. Accumulating studies indicated that exosomes serve as vehicles for virus transmission, but the role of exosomes in viral shedding through the semen remains unclear. In this study, exosomes purified from the REV-positive semen were shown with reverse transcription-PCR and mass spectrometry to contain viral genomic RNA and viral proteins, which could also establish productive infections both *in vivo* and *in vitro* and escape from the REV-specific neutralizing antibodies. More importantly, compared with the infection caused by free virions, the exosome is more efficient for the virus to ensure effective infection and replication, which can also help the REV compromise the efficacy of the host immune response. In summary, this study demonstrated that semen-derived exosomes can medicate the transmission and immune escape of REV, implicating a novel mechanism for REV entering the semen and leading to vertical transmission.

## Introduction

The first member of the reticuloendotheliosis virus (REV), including several closely related amphotropic avian retroviruses (family Retroviridae) (1), was isolated from a turkey in 1957 (2). After that, REV was reported in a diverse range of hosts including chicken (3), duck (4), geese (5), turkey (6), and wild birds (7). REV infection induces fatal hemangioblastoma, growth inhibition, and tumor formation in lymphatic tissue (8), which has caused huge economic losses in the world.

REV is an enveloped virus with a single-stranded positive-sense RNA molecule of approximately 8.4 kb, which is composed of the gag group-specific antigen (*gag*), polymerase (*pol*), and envelope (*env*) genes, as well as a long terminal repeat (LTR). At present, the extremely low genetic diversity observed among all avian REV isolates and sequences indicates a very recent origin for REV in birds (9). However, historical, phylogenetic, and paleovirological evidence totally supports a scenario wherein REVs originated as mammalian retroviruses (e.g., type C retroviruses) that were iatrogenically introduced into avian hosts (10). Besides, these integrated into the fowlpox virus (11, 12) and gallid herpesvirus-2 (13) genomes, and then generated the recombinant DNA viruses that now circulate in wild birds and poultry. Although antibodies to REV are widespread in poultry (14–18), the etiology of REV infection remains elusive (8). Recently, a study confirmed that the REV can enter the semen and transmit by artificial insemination, but how the virus gets into semen was still unclear (19).

Exosomes are a small subpopulation of extracellular vesicles secreted by most cell types which originate from a late endosomal compartment called “multivesicular bodies” (MVBs), existing in most biofluids, and are 30–150 nm in size (20). As a natural vehicle, various biological materials, including proteins, RNAs, and lipids, can be carried by exosomes (21), which mediate the cell-to-cell communication through the transmission of signaling-competent proteins or functional RNAs between cells (22–24). For viruses, the exosomes not only participated in viral replication, pathogenesis, and transmission (25), but also were vitally important to escape host immune response (26, 27). What is more, exosomes isolated from hepatitis C virus (HCV)-infected cells contain viral RNA and can mediate the viral-receptor-independent transmission of HCV (28). Thus, some viruses have evolved mechanisms to hijack the exosomes for immune escape and transmission, and these findings have increased our interest in the role of exosomes in virus entering the semen.

Therefore, semen-derived exosome was isolated, purified, and identified in this study, and systemic analysis was performed to show its biological characteristics as well as its function in mediating REV transmission and immune escape.

## Materials and Methods

### Cell Culture and Viruses

In this study, 9-day-old specific pathogen-free (SPF) chicken embryos were used to generate chick embryo fibroblast (CEF) cells, which were then maintained in DMEM (Invitrogen, USA) with 10% exosome-depleted fetal bovine serum (FBS, Thermo Fisher, USA) and 1% penicillin/streptomycin in a humidified incubator at 37°C with 5% CO_2_. The REV strain SDAUR-S1 (GenBank accession number: MF185397), which was isolated from the REV-positive cock semen in China (19), was used in this study. The viral titer was determined using the median tissue culture infective dose (TCID) method.

### REV-Positive Semen Collection

Sixty SPF chicken embryos (SPAFAS Poultry Company, China) were hatched until 9 days old and then inoculated with 10^3.5^ TCID_50_ SDAUR-S1 by yolk (group A), while 60 SPF chicken embryos hatched with the same condition served as control (group B). After being hatched, all chicks in each group were banded and separately bred in shielded cages with positive filtered air. Until chicks were 20 weeks of age, semen was collected from group A for REV detection using a published method (19). REV-positive samples were stored at −80°C for further analysis. Semen collected from group B was also analyzed by the REV detection to exclude the potential contamination.

### Exosome Isolation and Purification

REV-positive semen or control semen was diluted with equal phosphate buffered saline (PBS) and centrifuged for 5 min at 500 rpm to remove the sperm and larger debris. Then, the supernatant was transferred to a new tube and centrifuged at 2,500 rpm for 10 min at 4°C to further remove cell debris. The supernatant was then filtered serially through 1, 0.44, and 0.22 μm filters. After that, the exosome was isolated according to a published method (28). Briefly, the filtered supernatant for exosome isolation was concentrated to a final 1 ml volume using the Amicon Ultra15 Centrifugal Filter Unit with Ultracel-100 membrane (Millipore, USA). The concentrated filtered supernatant was used to isolate exosome by ExoQuick kits according to the specification of the manufacturer (System Biosciences, USA). Samples were gently mixed and incubated for 1 h at 4°C. Following incubation, exosomes were precipitated by centrifugation at 14,000 rpm for 10 min at 4°C. The precipitated exosomes were resuspended in 16 μl PBS. Purification of exosomes was done using anti-CD63 immunomagnetic capturing with primary anti-CD63 antibody (Thermo Fisher, USA) followed by corresponding secondary antibody coupled to magnetic beads (Miltenyi Biotec, Germany). The Miltenyi Biotec MidiMACS separator was used with LD columns for exosome purification.

### Electron Microscopy and Liquid Chromatography-Tandem Mass Spectrometry

Exosomes isolated by anti-CD63 immunomagnetic bead selection were resuspended in PBS and transferred to a formvar-coated copper grid (200 meshes) and then allowed to settle/attach for 30 min. The grid was washed by sequentially positioning droplets of PBS on top and using absorbing paper in between. The samples were then fixed by dropwise addition of 2% paraformaldehyde onto parafilm and placing the grid on top of the paraformaldehyde drop for 10 min. Fixation was followed by five washes with deionized water and samples contrasted by adding 2% uranyl acetate for 15 min. Afterward, the samples were embedded by adding a drop of 0.13% methyl cellulose and 0.4% uranyl acetate for 10 min. The grid was visualized using a transmission electron 423 microscope (Hitachi H-7000FA, Japan). For MS analysis, the purified exosomes were resuspended in 25 μl elution buffer (50 mM glycine, pH 2.8). Proteins were digested with the FASP procedure. Briefly, the protein pellet (about 30 μg) was solubilized in 30 μl SDT buffer 433 at 90°C for 5 min. The detergent, DTT, and other low-molecular-weight components were removed using 200 μl UA buffer (8 M urea, 150 mM Tris–HCl pH 8.0) with repeated ultrafiltration (Microcon-30kD Centrifugal Units). Iodoacetamide (0.05 M, 100 μl) in UA buffer was then added to block the reduced cysteine residues, and the samples were incubated for 20 min in the dark. The filter was washed three times with 100 μl of UA buffer and then twice with 100 μl of 25 mM NH_4_HCO_3_. Finally, the protein suspension was digested with 2 μg of trypsin (Promega) in 40 μl of 25 mM NH_4_HCO_3_ overnight at 37°C. The resulting peptides were collected as the filtrate. Experiments were performed on a Q Exactive mass spectrometer (Thermo Finnigan) coupled to an Easy-nLC1000 liquid chromatograph (Thermo, USA).

### Analysis and Quantification of REV RNA

For the detection of REV genome, RNA was isolated from the purified exosomes using a commercial kit (Bio-Tek, USA), and total RNA was resuspended in 12.25 µl of DNase-, RNase-, and proteinase-free water. According to the published sequence of SDAUR-S1 (19), five pairs of primers were designed to amplify the whole genome of REV by RT-PCR. Detailed information of those primers is shown in **Table 1**. These five downstream primers were also reverse transcription primers.

**Table 1:** Primers used for PCR amplification of the reticuloendotheliosis virus whole genome.

Fragments	Primer^a^	Sequence, 5′–3′	Position^b^
a	F1	AATGTGGGGAGCTCTGGGGGGAATA	1–25
	R1852	GTCGTGAACAGGGTACGGAG	1832–1852
b	F1776	GAATCCGTTTTCTACACACACCA	1776–1799
	R3892	CATTTCCCCCCCGCGTTGCCGCATA	3867–3892
c	F3813	ATTGGGTACTGCAGACTTTGGAT	3813–3838
	R5435	CCAAACCTGGGGATAATATCATGAA	5410–5435
d	F5352	TGGGTAGAGGAATATCCAGCGAGAA	5352–5377
	R7759	TCATGTATAATGCGAGTCAGGGTCT	7735–7759
e	F7060	TGTCCTCCACCAGGTCATGTATTTG	7060–7085
	R8471	AAATGTTGTACCGAAGTACT	8450–8471

a Primers F1 to R1852, F1776 to R3892, F3813 to R5435, F5352 to R7759, and F7073 to R8471 were used to amplify the overlapped five fragments a, b, c, d, and e of the whole reticuloendotheliosis virus (REV) genome; primers R1852, R3892, R5435, R7759, and R8471 are also reverse transcription primers.

b Positions of primers located in the complete genome are shown according to the REV semen strain SDAUR-S1.

To quantify the RNA copies of REV in REV-positive semen, REV exosome, REV-treated cells, or culture supernatant, the total RNA of those samples was extracted using the above method. Reverse transcription was performed with the AMV RT kit (TaKaRa, Japan). The primers used to quantify the REV were as follows: 5′-CCCCATTCATGTCCAGCTAT-3′ and 5′-AGGGAGGAGAGGAGTGTTCC-3′. Absolute real-time PCR was performed with the ABI 7500 PCR machine. Quantitative real-time PCR (qRT-PCR) reactions were performed in duplicate for each sample. All qRT-PCR reactions were performed with a no-template control.

### Proviral Load Quantification

Total DNA of the treated cells was extracted using a commercial kit (Bio-Tek, USA). The proviral load (PVL) was measured by qRT-PCR targeting REV and *HMG14b* (primers *HMG14b*-F and *HMG14b*-R) by Q SYBR green premix kit (TaKaRa) according to the protocol of the manufacturer. *HMG14b* is a known single-copy gene in the chicken genome and was thus used as a housekeeping reference gene (29). Thermal cycling conditions were 95°C for 5 min and 40 cycles each of 95°C for 5 s followed by 58°C for 30 s. The reactions were also performed on an ABI 7500 PCR machine (Thermo Fisher, USA).

### Exosome Infection Assays

CEF cells were seeded on 24-well plates at a density of 2 × 10^5^ cells/well. After determining the viral load, about 10^6^ genomic equivalents (GE) of REV-positive semen, REV exosome, free REV, and corresponding equal control semen and control exosome were incubated for 2 h at 37°C in 5% CO_2_ and then replaced with fresh maintenance medium. At 48 h post-infection (hpi), cell samples were fixed or collected for immunofluorescence assay (IFA), western blot (WB), and qRT-PCR analysis, respectively.

### Antibody-Mediated Neutralization of REV

REV-specific NAb was isolated and purified from the sera of SPF chickens repeatedly immunized with a published REV vaccine (30). The titer of purified NAb was adjusted to 1:32, as determined by using the neutralization assay. Specially, serial dilutions of antibodies were incubated with an equal volume of the REV semen strain SDAUR-S1 for 1 h at 37°C. The mixtures were added to 96-well plates containing CEF cells. After incubation for 48 h at 37°C in a humidified atmosphere containing 5% CO_2_, the cells were fixed for IFA assay according to the above methods. Neutralization titers were expressed as the reciprocal of the high dilution that inhibited at least 90% of the fluorescent foci present in the control wells. Next, REV-positive semen, REV exosome, free REV, and corresponding controls were incubated with the purified NAbs for 1 h, and then transferred into a monolayer of CEF cells. At 48 hpi, cell samples were fixed or collected for IFA, WB, and qRT-PCR analysis, respectively.

### Indirect Immunofluorescence Assay

Cells were fixed with precooled fixation fluid (acetone/alcohol, v/v, 3/2) for 8 min, then washed in PBS, and blocked with 3% bovine serum albumin (BSA) for 1 h. Next, cells were incubated with a REV-specific monoclonal antibody (mAb) 11B118 (31) for 45 min at room temperature. The cells were then stained with fluorescein isothiocyanate-conjugated goat anti-mouse antibodies (Invitrogen, USA), according to the protocol of the manufacturer. Finally, the cells were washed, and the nuclei were stained with DAPI (4′,6-diamidino-2-phenylindole) and mounted for confocal microcopy (Olympus FV1000, Japan).

### Western Blotting Analysis

Western blotting was performed with the following established protocol. Specifically, cell or exosome lysates were subjected to 12% SDS-PAGE electrophoresis and then transferred onto 0.2-μm polyvinylidene difluoride (PVDF) membranes (Millipore, USA). The membranes were blocked with Tris-buffered saline containing Tween 20 with 10% non-fat dry milk. The blots were then incubated with a primary antibody at 4°C overnight. The primary antibodies used were directed against CD63, CD9, Alix, GRP94 (Abcam, UK), and REV *pol*-*gag* polyprotein, *env*, and polymerase (made in our laboratory). The membranes were reprobed with β-actin as an interval control and then incubated with horseradish-peroxidase-conjugated secondary antibodies that included a goat anti-mouse IgG antibody or a goat anti-rabbit IgG antibody (Beyotime Biotechnology, China). Finally, the proteins were detected by using enhanced chemiluminescence (ECL, Bio-Rad).

### Inhibition of Semen-Derived Exosomes

GW4869 is a neutral sphingomyelinase inhibitor that is known to inhibit ceramide biosynthesis (32). In this study, the effects of GW4869 on semen-derived exosome releases and viral shedding were also examined to show the relationship between them. Specifically, 1, 2.5, 5, and 10 μmol GW4869 were injected into 10 cocks secreting the REV-positive semen intraperitoneally, respectively, whose semen was then collected after 48 h for WB, qRT-PCR analysis, and protein concentration determination using a commercial kit (Beyotime Biotechnology, China). Preliminary experiments revealed no obvious pathogenicity of any of the tested concentrations of GW4869 in chickens.

### Infectivity and Innate Immune Response of REV Exosome or Free Virion

To further compare the infectivity of different infections, REV exosome and free virions at the same genomic equivalent (10^6^) were used to infect the CEF cells, embryonated eggs (yolk-sac inoculation), and 1-day-old SPF chicks (intravenous injection), respectively. Samples collected at different intervals were used to determine the viral load and proviral load using the above methods, and EID_50_ assays were performed according to a published method (33). Meanwhile, using qRT-PCR, mRNA expressions of the innate immune-related genes in CEFs or livers were ascertained. The primers for chicken β-actin, TLR3, TLR7, MDA5, IL-1β, IL-6, IL-8, IFN-α, IFN-β, IFN-γ, OASL (Oligoadenylate synthase like protein), and Mx (Myxovirus resistance) genes were referenced to a previous study (34). REV exosome and free virions at the same genomic equivalent (10^6^) were also mixed into SPF semen for artificial insemination. Next, serum samples were collected from each hen to determine the REV-specific antibody using a commercial kit (IDEXX, USA), and cloacal cotton swabs were also collected for qRT-PCR analysis during 1–14 days after artificial insemination.

### Ethics Approval and Consent to Participate

The animal care and use protocol was approved by Shandong Agricultural University Animal Care and Use Committee (SDAUA-2016-002). All the experimental animals of this study were cared for and maintained throughout the experiments strictly following the ethics and biosecurity guidelines approved by the Institutional Animal Care and Use Committee of Shandong Agricultural University.

### Statistical Analysis

Statistical analysis was performed using the SPSS Statistical Software Package for Windows version 17.0 (SPSS Inc. Chicago, IL, USA). *p <*0.05 was considered statistically significant based on Duncan’s multiple-range test.

## Results

### Isolation, Identification, and Component Analysis of REV-Positive Semen-Derived Exosomes

**Figure 1:**
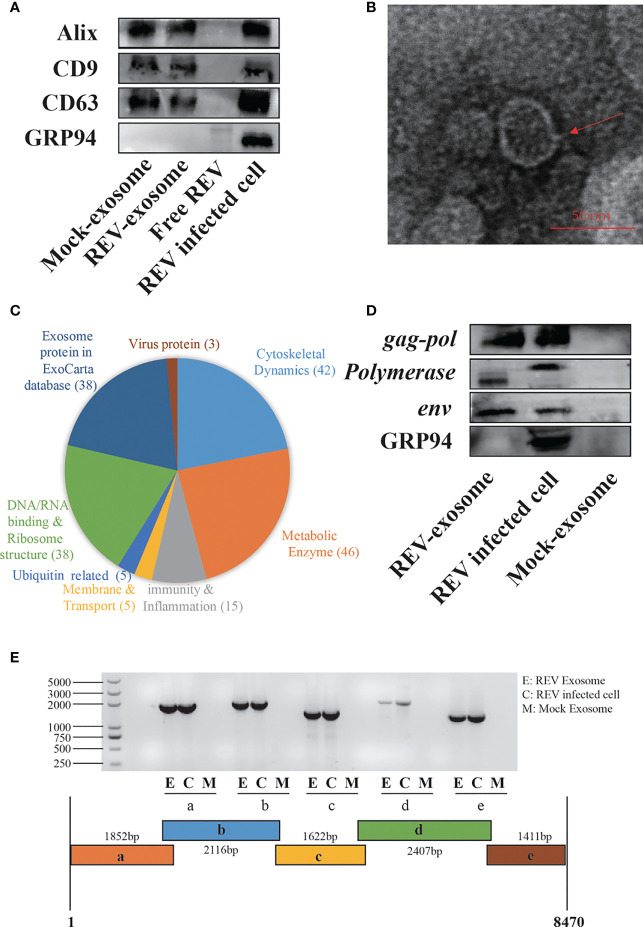
Isolation, characterization, and components analysis of exosomes derived from reticuloendotheliosis virus (REV)-positive cock semen. **(A)** Purified exosomes derived from mock- or REV-positive cock semen were analyzed on Western blots probed with antibody against Alix, CD9, CD63, and GRP94. Purified REV virions (free REV) and the REV-infected cell were used as controls. **(B)** Transmission electron microscopy observations of negatively stained purified exosomes from REV-positive cock semen; scale bar = 50 µm. **(C)** LC-MS/MS analysis of the purified exosome from REV-positive cock semen, classification of host proteins according to function if included. **(D)** REV proteins in exosomes were confirmed on Western blots probed with antibody directed against env, gag, and pol. **(E)** REV genomic RNAs in purified exosomes isolated from REV-positive cock semen were detected with RT-PCR. Five overlapping fragments were designed based on the genome sequence of REV strain SDAUR-S1.

For the establishment of cocks producing continuous REV-positive semen, 60 SPF embryos were inoculated with 10^3.5^ TCID_50_ REV semen strain SDAUR-S1 in yolk. The hatched cocks with chronic viremia as well as cocks in the control group were used for semen collection. REV virions and exosomes share similar sizes and buoyant densities, and the traditional ultracentrifugation and sucrose gradient isolation method is insufficient for isolating pure exosomes free of virus contamination. Therefore, an updated isolation method based on a commercial exosome isolation kit and CD63 immunomagnetic isolation was employed to extract and purify exosomes from REV-positive semen or mock semen (28). The purified exosomes were characterized by analysis of exosome markers using WB, and three representative exosome markers, Alix, CD9, and CD63, were all detected (**Figure 1A**). Transmission electron microscopy (TEM) showed that the purified exosomes displayed a typical cup-shaped appearance about 50 nm in size (**Figure 1B**). Furthermore, liquid chromatography-tandem mass spectrometry (LC-MS/MS) combined with Gene Ontology (GO) analysis was performed to characterize the proteins of semen-derived exosomes. A total of 192 proteins in different functional classification were identified within (**Figure 1C**). Also, LC-MS/MS revealed the presence of three REV-related proteins, namely, *pol*-*gag* polyprotein, *env*, and REV-polymerase, while WB analysis further confirmed it (**Figure 1D**). Consistent with previous studies suggesting that viral nucleic acids are present in exosomes from infected cells (35, 36), the REV whole-genome RNAs were also detected in REV-positive semen-derived exosomes (**Figure 1E**).

### Exosomes Transmit REV and Establish Productive Infections in CEF Cells

**Figure 2:**
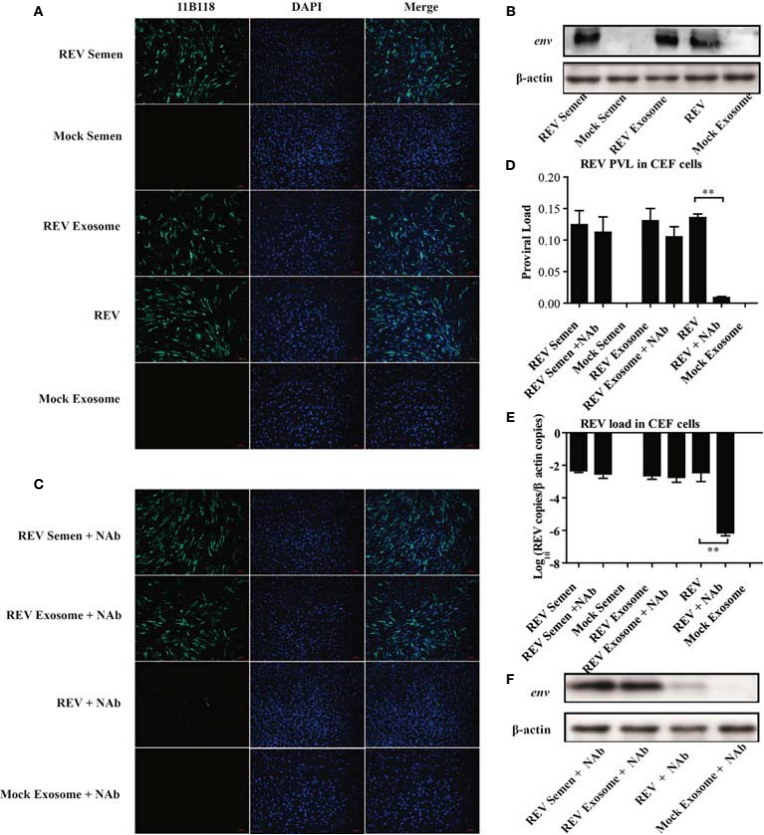
Semen exosomes transmit REV and establish productive infections in chick embryo fibroblast (CEF) cells. **(A)** Immunofluorescence assays (IFAs) demonstrated the productive infection of CEF cells after treatment with REV-positive semen or REV exosomes or infected with REV. At 48 hpi after treatment or infection, the cells were fixed and an IFA was performed with 11B118. **(B)** Western blotting analysis of env expression in CEF cells treated with REV semen or REV exosome or infected with REV. **(C)** REV-positive semen, REV exosome, or free REV was incubated with REV-specific NAbs for 1 h. Then, CEF cells were exposed to the antibody-treated exosomes or virus for 3 h. The semen, exosome, or virus was washed off and the medium was replaced with fresh maintenance medium for a further 48 h for IFAs. Quantitative RT-PCR was performed to detect REV proviral load **(D)** and viral load **(E)** in CEF cells. **(F)** Western blotting analysis of env expression in CEF cells treated with antibody-treated semen, exosome, or virus. **Indicates significant difference (*p* < 0.01) between the two experimental groups.

The observation of the REV whole genome and three viral proteins in the purified exosomes from REV-positive semen prompted us to verify its ability of transmitting infection. In this study, REV-positive semen (REV semen) and exosomes purified from it (REV exosome) were used to incubate CEF cells. IFA using REV-specific MAb 11B118 was performed at 48 hpi to confirm infection. Cells infected with cell-culture-derived REV (free REV) were used as the positive control, while cells inoculated with mock semen as well as exosomes isolated from mock semen (mock exosome) were used as the negative control. As shown in **Figure 2A**, the specific green fluorescence was clearly observed in the cells treated with REV semen, REV exosome, and free REV, which demonstrated that both the REV semen and REV exosome could establish a productive infection in the CEF cells. The expression of viral protein *env* could also be detected using WB in the above CEF cells, which further confirmed the establishment of productive REV infections (**Figure 2B**).

This study also investigated whether semen or exosome-mediated REV transmission is blocked by REV-specific NAbs. As expected, there was almost no difference between the REV semen/exosome group and the REV semen/exosome + NAbs group (**Figure 2C**). However, the number of infected cells decreased significantly in the group treated with free REV and NAbs than in the group treated with free REV without NAbs (**Figure 2C**). These results indicated that NAbs efficiently neutralized free REV infection but had almost no blocking effect on REV semen or exosome-mediated transmission. A similar result was also confirmed with WB analysis of the expression of the viral *env* protein (**Figure 2F**). qRT-PCR analysis was performed to determine the REV PVL and viral load in those groups, and the results also support the above conclusions. Specifically, both high PVL and viral titer were generated in CEF cells treated with REV semen and REV exosome, which was similar to the levels seen in cells infected with the free REV. REV-specific NAbs significantly inhibited the infection and replication of free REV in CEF cells but did not make much difference to REV semen or REV exosome (**Figures 2D, E** **)**. Taken together, these results suggest that both REV semen and purified REV exosomes can establish productive infections in CEF cells and ignore the antibody neutralization.

### Inhibition of Exosome Release Decreased the REV Viral Load in Semen

**Figure 3:**
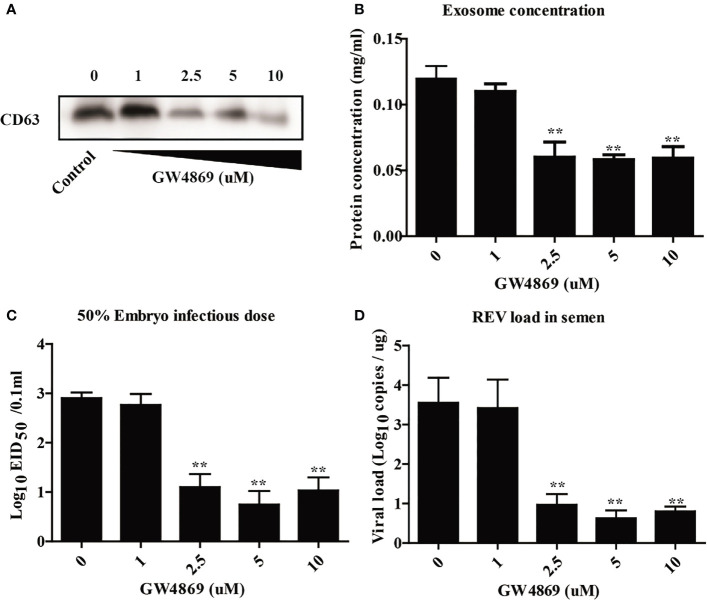
Inhibition of exosome release decreased the viral load of REV in semen. **(A)** The isolated exosomes from cocks treated with GW4869 in different doses were subjected to Western blotting analysis of CD63 expression. **(B)** Protein concentration analysis using the BCA kit. **(C)** The viral titers were determined with the EID_50_ assay. **(D)** Quantitative RT-PCR was performed to detect REV viral load in isolated exosomes. **Indicates significant difference (*p* < 0.01) between the two experimental groups.

To further investigate the relationship between the semen-derived exosome and REV viral shedding, the effect of an inhibitor of exosome releases, GW4869, on viral shedding in semen was also determined in this study. The effect of GW4869 on exosome biogenesis was assessed with WB to quantitate the expression of exosome biomarker CD63 (**Figure 3A**), exosome protein concentration analysis (**Figure 3B**), EID_50_ assays (**Figure 3C**), and viral load quantitation (**Figure 3D**). Results showed that the amount of exosome significantly declined in semen from cocks treated with GW4869 (2.5, 5, and 10 μmol, **Figures 3A, B** **)**. Meanwhile, being consistent with the change of the exosome releases, the infectivity or the REV viral load of the semen dropped, too (**Figures 3C, D** **)**. All those results demonstrated that there is a positive correlation between REV viral load and the amount of exosome in semen.

### Exosome Showed Higher Transmission Efficiency Than Free Virions

**Figure 4:**
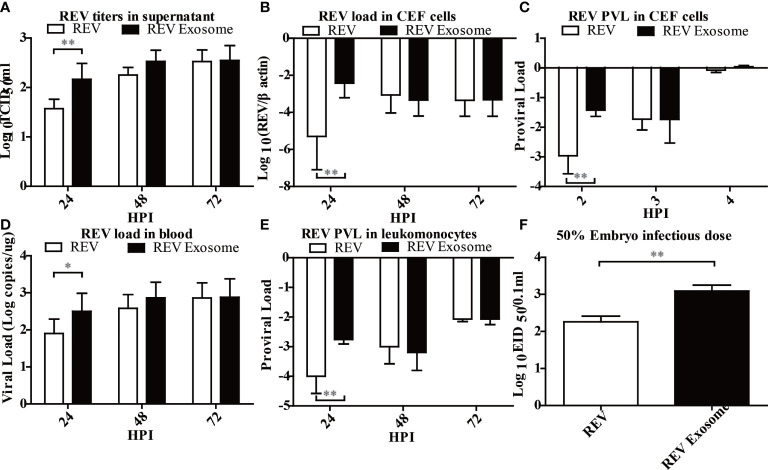
Infection ability analysis of the REV exosome and free REV both *in vivo* and *in vitro*. **(A)** REV titers in supernatant of CEF cells treated with free REV and REV exosomes (10^6^ genomic equivalents) at 24, 48, and 72 hpi, and the viral titers harvested at different intervals were calculated and expressed as TCID_50_ per milliliter. **(B)** REV load in CEF cells treated with free REV and REV exosomes (10^6^ genomic equivalents) at 24, 48, and 72 hpi, and REV viral load levels were normalized to β-actin. **(C)** REV proviral load in CEF cells treated with free REV and REV exosomes (10^6^ genomic equivalents) at 2, 3, and 4 hpi, and REV proviral load levels were normalized to *HMG14b*. **(D)** REV viral load in the blood of SPF chicks treated with free REV and REV exosomes (10^6^ genomic equivalents) at 24, 48, and 72 hpi, and viral RNA concentration (log10) were normalized per 1 µg of total RNA. **(E)** REV proviral load at 24, 48, and 72 hpi were determined by the presence of REV-cDNA in the leukomonocytes of SPF chicks treated with free REV and REV exosomes (10^6^ genomic equivalents), and REV proviral load levels were normalized to *HMG14b*. **(F)** The viral titers of free REV and REV exosomes (10^6^ genomic equivalents) were determined with the EID_50_ assay. *Indicates significant difference (p < 0.05) between the two experimental groups. **Indicates significant difference (*p* < 0.01) between the two experimental groups.

Previous studies showed that exosomes can transmit virus and establish productive infections (28, 37), but there was no report about the transmission efficiency of exosomes. To this end, the transmission efficiency of exosomes or free virion was determined using CEF cells, chicks, and embryonated eggs. As shown in **Figure 4**, the virus titer in the supernatant (**Figure 4A**), the viral load (**Figure 4B**), and the PVL (**Figure 4C**) in CEF cells treated with REV exosomes were all significantly higher than those of free REV at the early stage of infection. Similarly, the viral load in the blood (**Figure 4D**) and the PVL (**Figure 4E**) in chicks treated with REV exosome were also significantly higher than those of the free REV at 2 and 24 hpi, respectively. What is more, the ability of REV exosomes to infect embryonated eggs was also significantly higher than that of free REV determined by EID_50_ assays (**Figure 4F**).

**Figure 5:**
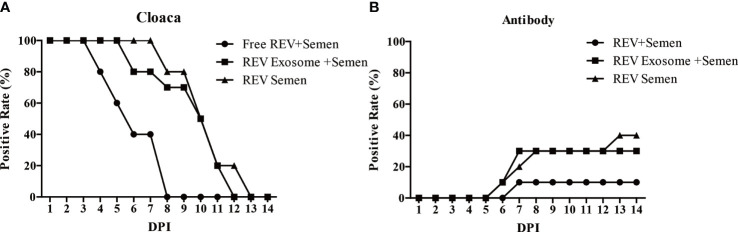
Effects of free REV and REV exosome on hens by artificial insemination. **(A)** REV-positive rate of cloaca after artificial insemination during 1 to 14 dpi. **(B)** REV-antibody-positive rate of hens after artificial insemination during 1 to 14 dpi.

The transmission efficiency of REV exosomes or free virions in hens by artificial insemination was also examined in this study. Results showed a similar transmission efficiency of REV-positive semen and mock semen + REV exosome. The cloaca or antibody-positive rate was higher than that of mock semen + free virions group (**Figures 5A, B** **)**. These results suggested that exosomes survive more easily in the cloaca and have a stronger ability to infect hens than free virions.

### Exosome-Mediated REV Infection Induced Lower Innate Immune Response Than Free Virions in Infected CEF Cells and Chicks

**Figure 6:**
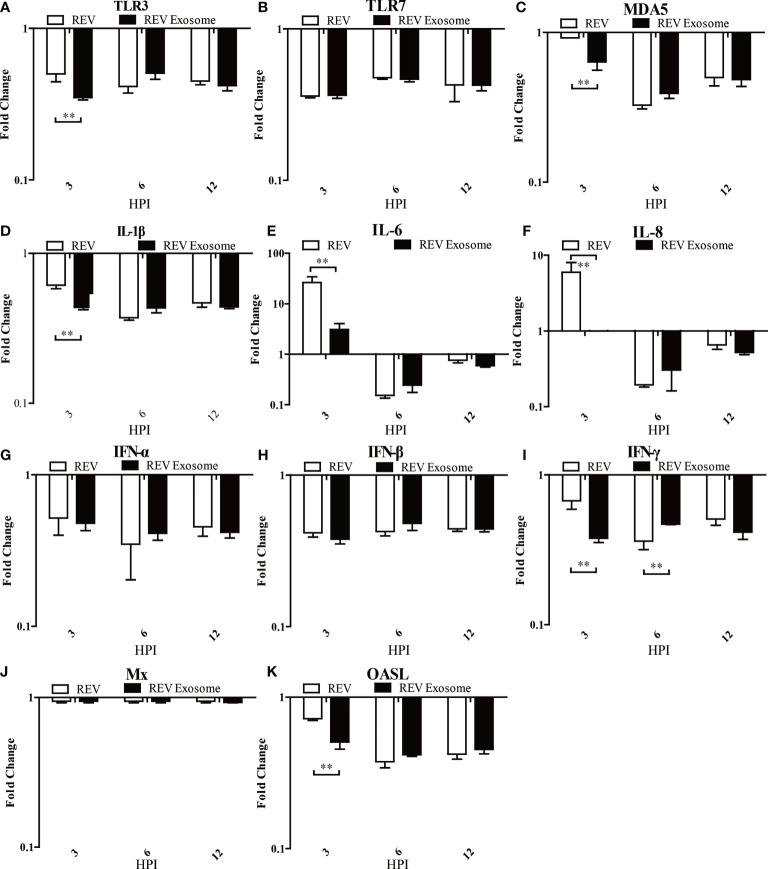
Expression profiles of immune-related genes in infected CEF. Cells were infected with equal REV or REV exosome and then collected at 3, 6, and 12 hpi. The inducible gene expression levels in the cells were tested using real-time quantitative reverse transcription polymerase chain reaction analysis. Fold change was calculated by comparing the experimental group with controls at the same sampling point, and relative expression levels were normalized to the β-actin gene and calculated using the 2^−△△Ct^ method. Data are the means across three independent experiments, and each experiment was analyzed in triplicate. Results are expressed as means ± SDs (*n* = 3). **Indicates significant difference (*p* < 0.01) between the two experimental groups. **(A–K)** Expression levels of TLR3, TLR7, MDA5, IL-1β, IL-6, IL-8, IFN-α, IFN-β, IFN-γ, OASL, and Mx in CEFs.

To identify which pattern recognition receptors (PRRs), cytokines, and IFN-stimulated genes could be involved in the antiviral innate immune response to REV infection induced by exosomes or free virions, mRNA expressions of TLR3, TLR7, MDA5, IL-1β, IL-6, IL-8, IFN-α, IFN-β, IFN-γ, OASL, and Mx were examined. In CEF cells, mRNA expressions of TLR3, TLR7, MDA5, and IL-1β showed sustained downregulation at 3, 6, and 12 hpi, but the mRNA expressions of TLR3, MDA5, and IL-1β in the REV group were higher than those of the REV exosome group (**Figures 6A–D**). The mRNA expressions of IL-6 and IL-8 showed similar trends, both significantly increasing at 3 hpi and returning to downregulation at 6 and 12 hpi, while their mRNA expressions in the REV group were also higher than that of the REV exosome group (**Figures 6E, F** **)**. What is more, decreases were also seen in IFN-α and IFN-β mRNA expression levels, and no significant difference appeared between those two experimental groups (**Figures 6G, H** **)**. The mRNA expressions of IFN-γ and OASL were also downregulated in CEF cells treated with REV exosomes or free virions, but their mRNA expressions in the former were lower than those of the latter at 3 hpi (**Figures 6I, K** **)**. No obvious upregulation or downregulation was found in Mx mRNA expression (**Figure 6J**).

**Figure 7:**
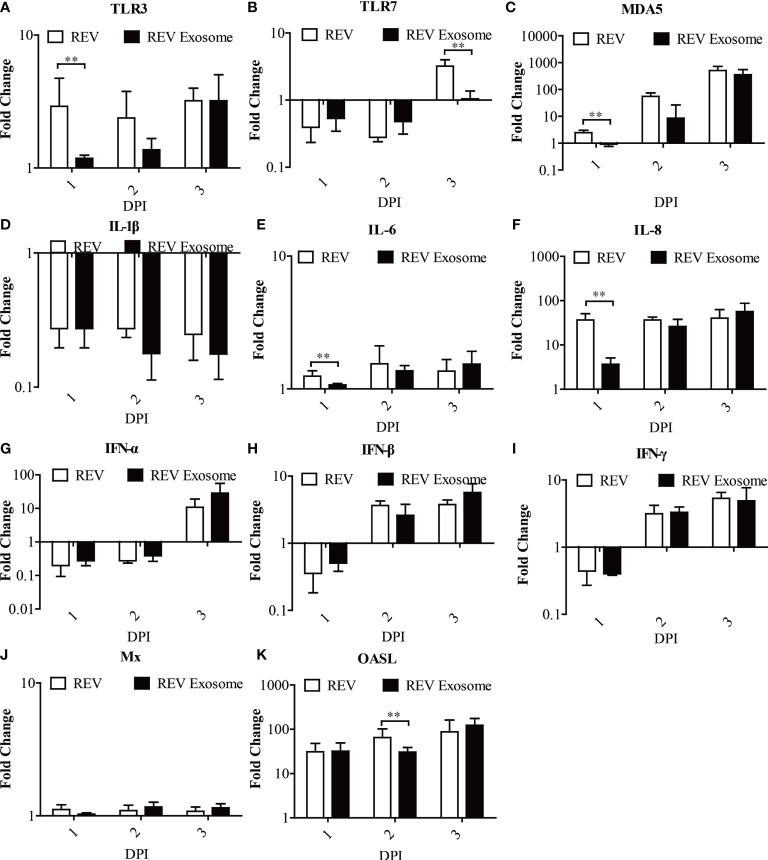
Expression profiles of immune-related genes in the livers from infected chicks. Liver samples from chickens were collected at 1, 2, and 3 dpi. The inducible gene expression levels in the cells were tested using real-time quantitative reverse transcription polymerase chain reaction analysis. Fold change was calculated by comparing the experimental group with controls at the same sampling point, and relative expression levels were normalized to the β-actin gene and calculated using the 2^−△△Ct^ method. Data are the means across three independent experiments, and each experiment was analyzed in triplicate. Results are expressed as means ± SDs (*n* = 3). **Indicates significant difference (*p* < 0.01) between the two experimental groups. **(A–K)** Expression levels of TLR3, TLR7, MDA5, IL-1β, IL-6, IL-8, IFN-α, IFN-β, IFN-γ, OASL, and Mx in infected chicks.

To further study exosome-induced innate immune responses, 1-day-old chicks were infected. At 1, 2, and 3 dpi, livers were collected from three chicks in each group for gene expression analysis (**Figures 7A–K**). In chicks, mRNA expression levels of TLR3, MDA5, IL-6, and IL-8 in those two experimental groups were all significantly upregulated at 1, 2, and 3 dpi, but the transcript levels of the REV group were higher than those of the REV exosome group at 1 dpi (**Figures 7A, C, E, F** **)**. The mRNA expressions of TLR7 decreased at 1 and 2 dpi and returned to upregulation at 3 dpi, while its mRNA expressions in the REV group were also significantly higher than those of the REV exosome group at 3 dpi (**Figure 7B**). In addition to this, the mRNA expressions of IFN-α, IFN-β, and IFN-γ showed similar trends, both significantly increasing at 1 dpi and gradually returning to downregulation at 2 and 3 dpi, and no significant difference appeared between those two experimental groups (**Figures 7G–I**). Being similar to that of *in vivo* experiments, the mRNA expression levels of Mx in chicks showed no obvious upregulation or downregulation (**Figure 7J**). What is more, the mRNA expression levels of OASL in those two experimental groups were all significantly upregulated at 1, 2, and 3 dpi, but the transcript levels of the REV group were higher than those of the REV exosome group at 2 dpi (**Figure 7K**).

## Discussion

REV is the etiological agent of one of the most important immune-suppression diseases with a significant economic burden worldwide. Epidemiology investigation showed that REV is highly prevalent in poultry, but the etiology for it remains elusive (14–18). Recently, REV was verified that it can enter the semen and transmit by artificial insemination (19), which is consistent with the human immunodeficiency virus (HIV) (38) or Zika virus (ZIKV) (39). Exosomes were 30–100 nm vesicles in most biofluids and can transfer functional cellular proteins, RNAs, and miRNAs; participate in intercellular communication; regulate cell functions; and even play an important role in viral pathogenesis and spreading (20). Thus, we speculated that the exosome might be the vehicle for virus entering the semen.

At present, several studies paid close attention to exosomes in different biofluids and made some achievements (20), but rare reports mentioned semen-derived exosomes, especially its function in viral spreading. Drawing on the experience of previous studies (28), semen-derived exosomes were isolated and purified with a commercial exosome isolation kit and CD63 immunomagnetic isolation method. WB analysis was used to confirm the existence of exosomes in semen. Furthermore, LC-MS/MS and RT-PCR analysis showed the presence of viral proteins and whole-genome RNA in purified REV exosomes. Moreover, *in vivo* experiments verified that exosomes can transmit REV and establish productive infections in CEF cells. More importantly, this study showed that REV-positive semen or exosome-mediated REV transmission is also resistant to REV-specific NAbs compared with the free virus, which is consistent with earlier observations in exosome-mediated transmission of HAV and enterovirus 71 (26, 27). All these results suggested that viral exosomes can escape humoral immune response. However, current results cannot determine whether the exosomes contain intact virus particles or infectious virus fragments, which needs further research.

An increasing number of studies explored the composition changes of exosomes after viral infection as well as its function in virus spreading (25, 40–43). In this study, the infections with exosomes compared with the free virion showed a tendency for higher levels of REV transmission, which leads to a higher infection rate and replication rate in the early stage of infection. Besides, the REV exosome has a higher infectivity in embryonated eggs or hens after artificial insemination. All these results suggested that the exosome-mediated REV transmission is more efficient, but the mechanism behind this needs further analysis.

On the other hand, the first line of defense against microbial infection is the innate immune response, which plays an important role in recognizing and killing the pathogens in the early stage of infections. Thus, the effects of the innate immune system on exosome-mediated viral infection have also attracted our interest. Interestingly, similar patterns of mRNA expression levels of innate immune-associated genes appeared in REV exosome or free virion-mediated viral infections, but the mRNA expression of pattern recognition receptors, including TLR3 and MDA5, and interleukin (IL) like IL-6 and IL-8 of REV exosome-mediated viral infections was significantly higher than that of free virion-mediated infection both *in vivo* and *in vitro*. These results demonstrated that, compared with the free virions, the exosome-mediated viral infection was less likely to be recognized by the innate immune system, leading to a lower cellular immunity level in the early stage of infection and then decreasing the barrier in REV exosome infecting the host.

In conclusion, our novel findings provide mechanistic insights into how semen-deprived exosomes mediate the transmission of REV, which is a clever strategy by the virus to ensure effective infection and help the REV escape the host immune responses.

## Data Availability Statement

The original contributions presented in the study are included in the article/supplementary material. Further inquiries can be directed to the corresponding author.

## Ethics Statement

The animal study was reviewed and approved by Shandong Agricultural University Animal Care and Use Committee.

## Author Contributions

QS and YZ conceived and performed the experiments, analyzed the data, and drafted the manuscript. ZC and PZ supervised the project and edited the manuscript. SC conducted part of the experiments. All authors contributed to the article and approved the submitted version.

## Funding

This study was supported by the National Key Research and Development Program of China (grant number 31972663).

## Conflict of Interest

The authors declare that the research was conducted in the absence of any commercial or financial relationships that could be construed as a potential conflict of interest.

## Publisher’s Note

All claims expressed in this article are solely those of the authors and do not necessarily represent those of their affiliated organizations, or those of the publisher, the editors and the reviewers. Any product that may be evaluated in this article, or claim that may be made by its manufacturer, is not guaranteed or endorsed by the publisher.
